# Asthma Powders

**Published:** 1907-07-06

**Authors:** James Sawyer

**Affiliations:** Birmingham


### Editor's Letter-Box.

### ASTHMA POWDERS.

Sir,—I have published several formulæ for asthma powders, and I have read with much interest your useful article upon such medicaments in the issue of The Hospital for June 15. There is, however, a sentence in that article which seems to be in need of qualification. It is, "Almost all asthma powders contain potassium nitrate, the purpose of which is simply to ensure a continuous lively smouldering of the lighted mixture, with evolution of abundant fumes; most of the powders contain either lobelia or stramonium, or both; these are the source of the active anti-spasmodic principle given off in the fumes." Now it appears that the fumes produced by the deflagration of saltpetre with vegetable fibre are prominent amongst the active anti-spasmodic principles which are given off in the smoke of the burning of an asthma powder containing that salt. The efficacy of such fumes, as used with benefit in spasmodic asthma, and as employed alone in the burning of touch paper, has long been recognised by the profession, and were described by Pereira under the names of inhalatio nitrosa and fumigatio nitrosa more than fifty years ago.

Yours faithfully,

James Sawyer.

Birmingham, June 25, 1907.

